# *CD44* deficiency inhibits unloading-induced cortical bone loss through downregulation of osteoclast activity

**DOI:** 10.1038/srep16124

**Published:** 2015-11-04

**Authors:** Yuheng Li, Guohui Zhong, Weijia Sun, Chengyang Zhao, Pengfei Zhang, Jinping Song, Dingsheng Zhao, Xiaoyan Jin, Qi Li, Shukuan Ling, Yingxian Li

**Affiliations:** 1 State Key Lab of Space Medicine Fundamentals and Application, China Astronaut Research and Training Center, Beijing, China; 2 Key Laboratory of Molecular and Cellular Biology of Ministry of Education, College of Life Science, Hebei Normal University, Shijiazhuang, China

## Abstract

The CD44 is cellular surface adhesion molecule that is involved in physiological processes such as hematopoiesis, lymphocyte homing and limb development. It plays an important role in a variety of cellular functions including adhesion, migration, invasion and survival. In bone tissue, CD44 is widely expressed in osteoblasts, osteoclasts and osteocytes. However, the mechanisms underlying its role in bone metabolism remain unclear. We found that CD44 expression was upregulated during osteoclastogenesis. *CD44* deficiency *in vitro* significantly inhibited osteoclast activity and function by regulating the NF-κB/NFATc1-mediated pathway. *In vivo*, *CD44* mRNA levels were significantly upregulated in osteoclasts isolated from the hindlimb of tail-suspended mice. *CD44* deficiency can reduce osteoclast activity and counteract cortical bone loss in the hindlimb of unloaded mice. These results suggest that therapeutic inhibition of CD44 may protect from unloading induced bone loss by inhibiting osteoclast activity.

CD44 participates in diverse signaling pathways ranging from growth factor-induced signaling to its ligand mediated pathways. Increasing evidence demonstrates that CD44 acts as a signaling hub controlling cell surface receptors of very diverse structure and function^1^,^2^. These receptors, through interactions with their principal ligands, provide bone cells with the ability to sense changes in the extracellular environment^3^,^4^,^5^,^6^. The macromolecules hyaluronan (HA), osteopontin (OPN), fibronectin, and collagen I can bind to CD44 and activate intracellular signaling^7^,^8^,^9^,^10^. These ligands are important regulators of bone remodeling. OPN knockout (KO) mice are resistant to hindlimb unloading and ovariectomy-induced bone loss^11^,^12^. However, the Roles of CD44 in the regulation of bone homeostasis remain unclear.

CD44 plays diverse roles in promoting pre-osteoclast fusion^13^, and specific CD44 antibody inhibits osteoclast formation^14^,^15^. The fusion of macrophages is inhibited by the binding of CD44 ligands OPN and HA^16^,^17^,^18^. CD44 is activated by MMP9, which leads to proteolytic cleavage of CD44 and produces an intracytoplasmic domain called CD44-ICD^19^,^20^, which binds to Runx2 and activates the expression of many genes. This domain can also promote the fusion of macrophages^13^,^21^. Galectin-9 induces osteoblast differentiation through the CD44/Smad signaling pathway^22^. Osteoclasts express CD44, and the interplay of CD44 with extracellular matrix proteins such as OPN may regulate osteoclast function^8^,^10^,^23^,^24^,^25^,^26^. However, little is known regarding to the mechanism underlying CD44-mediated osteoclast activity. *CD44*-deficient mice are viable without obvious developmental defects and show no overt abnormalities^27^. The changes in bone phenotype of *CD44* KO mice under hindlimb-unloading conditions have not been previously reported.

In this study, we found that CD44 expression was clearly up-regulated during M-CSF and RANKL-induced osteoclastogenesis. The activity and function of osteoclasts were significantly reduced in *CD44-*deficient mice via downregulation of the NF-κB/NFATc1 pathway. In addition, *CD44* mRNA levels were specifically upregulated in osteoclasts from hindlimb-unloaded mice, and cortical bone loss was ameliorated in *CD44* KO mice in this model, via downregulation of osteoclast function rather than by changes in osteoblast function.

## Results

### *CD44* deficiency inhibits osteoclastogenesis

**Figure 1:**
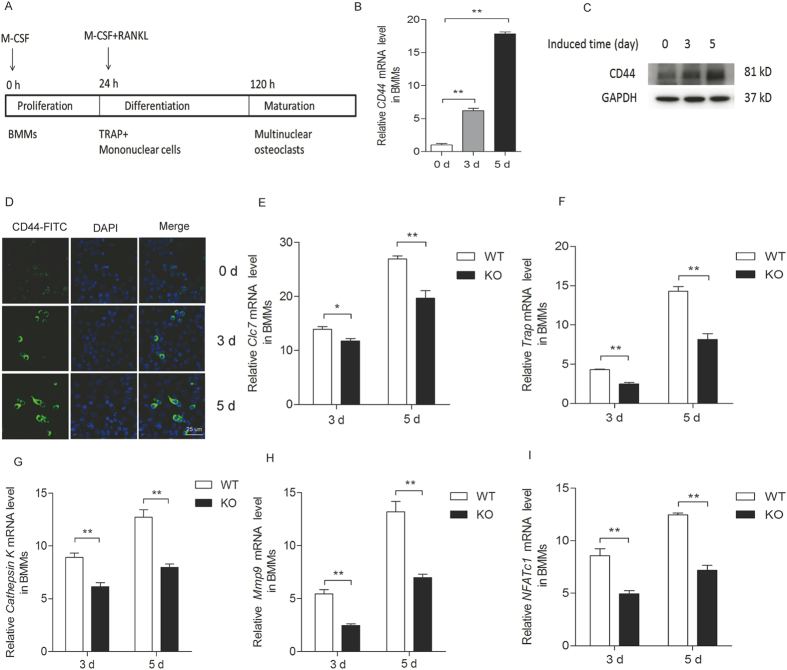
*CD44* deficiency inhibits the osteoclast differentiation of BMMs *in vitro*. (**A**) Schematic presentation of BMMs cultures, BMMs from two-month-old WT and *CD44* KO mice were cultured in medium with M-CSF (30 ng/ml) and RANKL (50 ng/ml) for 5 days. (**B**) The CD44 mRNA level and (**C**) protein level was determined in the process of osteoclast differentiation of WT BMMs by qPCR. The expression level was normalized to *Gapdh*. Data are the mean ± SEM. n=3; **p < 0.01, compared to 0 day. (**D**) Immunofluorescence for CD44 (green) in the process of osteoclastogenesis on day 0, 3 and 5. (**E-I**) The mRNA levels of *Clc7*, *Trap*, *CathepsinK*, *Mmp9* and *NFATc1* in WT and *CD44* KO BMMs were analyzed by qPCR. The transcripts levels were normalized to *Gapdh*. All data are the mean ± SEM. n=3, *p < 0.05, **p < 0.01, compared to WT.

Bone marrow monocytes (BMMs) isolated from bone marrow cells were induced into osteoclasts in the presence of M-CSF (30 ng/mL) and RANKL (50 ng/mL) (Fig. 1A). To investigate the potential role of CD44 in this process, we examined the changes of its mRNA and protein levels during osteoclastogenesis, and found that they progressively increased during this process. Specifically, *CD44* mRNA levels increased 6-fold on day 3 after induction, and reached 17-fold on day 5 compared to day 0 (Fig. 1B). CD44 protein levels were also significantly increased during osteoclastogenesis (Fig. 1C, see Supplementary Fig. S1A online). Immunofluorescence for CD44 showed the same results (Fig. 1D). When comparing the osteoclast differentiation potential of BMMs from wild-type (WT) and *CD44*-deficient (KO) mice, the expression levels of molecular marker genes for osteoclast function, including *Clc7*, *Trap*, *CathepsinK*, and *Mmp9*, were significantly reduced during osteoclast differentiation (Fig. 1E–H), and the transcription factor NFATc1, which plays a critical role in osteoclast differentiation, was also decreased (Fig. 1I). Western blotting results also revealed a much lower levels of NFATc1 and TRAP5 in osteoclasts from *CD44* KO mice than that from WT mice (see Supplementary Fig. S1B online). These results indicate that CD44 plays an important role in the process of osteoclastogenesis.

### Loss of *CD44* decreases osteoclast function

**Figure 2:**
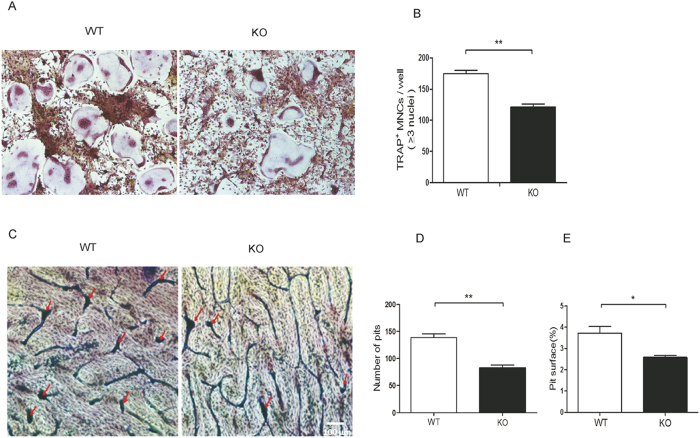
*CD44* deficiency inhibits osteoclastogenesis. (**A**) BMMs were isolated from WT and *CD44* KO mice, cultured in the presence of M-CSF (30 ng/ml) and RANKL (50 ng/ml) for 5 days. TRAP-stained cells show the fusion of BMMs on day 5. (**B**) The numbers of TRAP^+^ OCs with three or more nucleis are shown. To quantify the number of TRAP-stained cells in BMMs, at least 3 wells of each experiment per group were captured with a digital camera and counted. The number of experiment per group is 3. *p < 0.05, **p < 0.01, compared to WT. **p < 0.01, compared to WT. (**C**) Toluidine blue staining shows the eroded area of bone resorption. WT and *CD44* KO BMMs were cultured in OC medium for 5 days. Then OCs were cultured on bovine bone slices with OC medium for 2 days. The pits formation (arrows in red) was shown. (**D**) The numbers and (**E**) areas of bone resorption pits on bovine bone slices were measured by image analysis. To quantify the number and areas of bone resorption pits in bovine bone slices, 10 fields at 100 × magnification of each slices were counted, and at least 3 slices of each group were captured with a digital camera and analyzed using Image Pro 405 Plus 6.2 software (Media Cybernetics Inc. USA). *p < 0.05, **p < 0.01, compared to WT.

To investigate the effect of *CD44* deficiency on osteoclast function, we compared the changes of osteoclast fusion and bone resorption ability of osteoclasts with *CD44* KO or not. BMMs were cultured in the presence M-CSF (30 ng/mL) and RANKL (50 ng/mL) for 5 days (Fig. 1A), after which the number of TRAP-positive, multinucleated osteoclasts per well were counted. The number of multinucleated osteoclasts was remarkably decreased by nearly 50% in the *CD44* KO group (Fig. 2A,B). Mature osteoclasts can absorb bone surface. When we cultured these osteoclasts on bovine bone slice for 2 days, we observed pit formation by toluidine blue staining. Consistent with the result of TRAP staining, the number of pits and the eroded area of bone resorption were significantly decreased in the bovine bone slices cultured with *CD44* KO osteoclasts (Fig. 2C,D,E). These results demonstrate that *CD44* deficiency inhibits osteoclasts function.

### CD44 regulates osteoclast differentiation through the NF-κB signaling pathway

**Figure 3:**
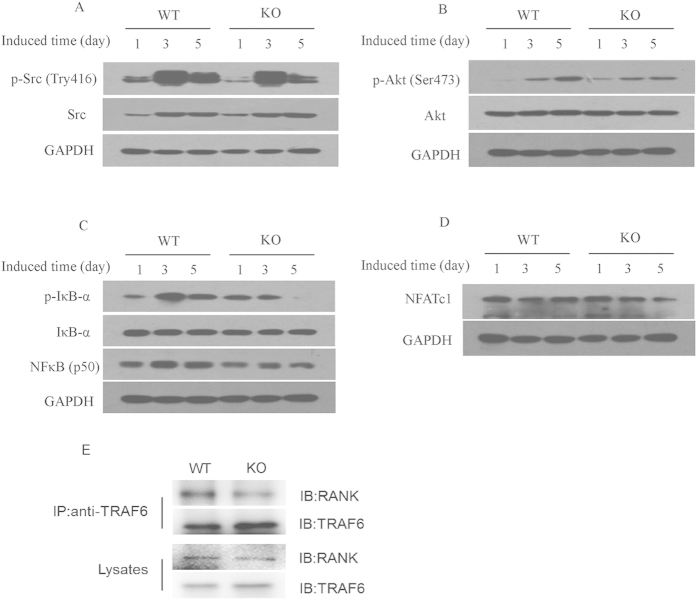
CD44 regulates osteoclast differentiation through NF-κB signaling pathway. BMMs from two-month-old WT and *CD44* KO mice were cultured with M-CSF (30 ng/ml) and RANKL (50 ng/ml) for 1, 3, and 5 days. Cell lysates were subjected to western blot analysis using specific antibodies. Representative western blot of p-Src (Tyr416) and c-Src (**A**), p-Akt (S473) and Akt (**B**), p-IκBα, IκBα and NF-κB (**C**), NFATc1 (**D**) were shown. GAPDH was used as internal control. (**E**) The effect of CD44 on the interaction between TRAF6 and RANK. BMMs from two-month-old WT and *CD44* KO mice were cultured with M-CSF (30 ng/ml) and RANKL (50 ng/ml) for 5 days, the cell lysate was immunoprecipitated by TRAF6 antibody, followd by RANK detection with anti-RANK antibody.

The NF-κB signaling pathway plays an essential role in osteoclast differentiation, function, and survival^28^,^29^,^30^,^31^. After binding to RANKL, RANK recruits TRAF6 to activate signaling cascades controlling osteoclastogenesis^32^,^33^,^34^, which include the phosphorylation of Src and Akt and the activation of NF-κB. The phosphorylation of Src can also enhance NF-κB activity via stimulation of Akt and IκB kinase activity. To investigate the regulation of CD44 on the signaling pathway related to osteoclast differentiation, BMMs from WT and *CD44* KO cells were cultured with M-CSF and RANKL for 1, 3 and 5 days, after which western blotting was used to analyze the expression of Src, Akt, and NFκB expression during osteoclast differentiation. As shown in Fig. 3A,B and see Supplementary Fig. S2A online, both p-Src (Tyr416) and p-Akt (Ser473) levels in WT BMM were higher than those in *CD44* KO mice. After induction with M-CSF and RANKL, p-IκB-α levels were increased during osteoclastogenesis, but significantly decreased in *CD44* KO cells. NF-κB protein levels peaked at day 5, and were much higher than those in *CD44* KO cells (Fig. 3C and see Supplementary Fig. S2A online). Next, we examined the protein levels of the osteoclastogenic transcription factor NFATc1, which was downstream of NF-κB, and found they were upregulated during osteoclast differentiation. However, its levels were significantly decreased in *CD44* KO cells (Fig. 3D and see Supplementary Fig. S2A online). During RANKL-induced osteoclastogenesis, TRAF6, can be directly recruited into RANK cytoplasmic domains and triggers downstream signaling molecules for the activation of Src, Akt and NF-κB. To explore whether CD44 can influence the interaction of TRAF6 with RANK, we performed coimmunoprecipitation experiment in RANKL-induced WT and *CD44* KO BMMs. The results showed that CD44 could promote the interaction between TRAF6 and RANK (Fig. 3E and see Supplementary Fig. S2B online). We also found the CD44 ligand, HA or OPN, could not activate the Src, Akt, and NFκB signal in these cells (see Supplementary Fig. S2C, D online). These results demonstrate that the NF-κB signaling pathway is inhibited during osteoclast differentiation of *CD44* KO BMMs, and suggest that CD44 regulates osteoclast differentiation through regulating RANKL-RANK mediated NF-κB signaling pathway.

### *CD44* deficiency suppresses hindlimb unloading-induced cortical bone loss

**Figure 4:**
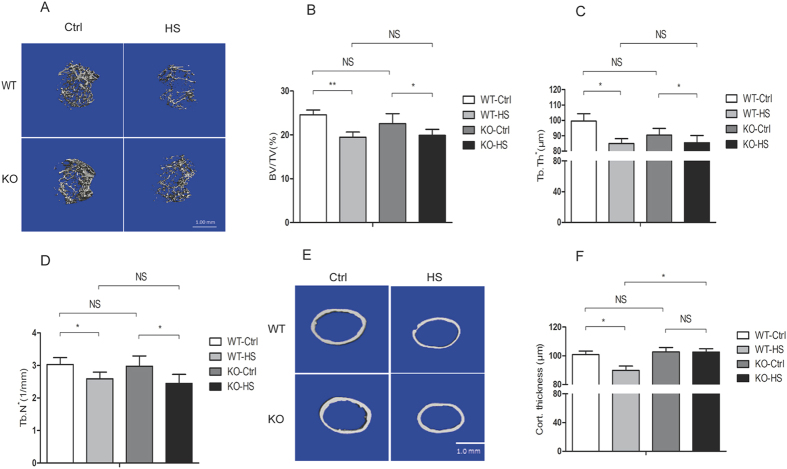
*CD44* deficiency suppresses hindlimb unloading-induced bone loss. (**A**) The 4-month-old WT and *CD44* KO mice were subjected to hindlimd unloading through tail suspension for 28 days, μCT images of proximal femurs from WT-control (WT-Ctrl, n=6), WT-hindlimb-unloading (WT-HS, n=6), *CD44* KO-control (KO-Ctrl, n=8) and KO-hindlimb-unloading (KO-HS, n=8) mice were shown. Trabecular bone volume per total volume (BV/TV %) (**B**), Trabecular thickness (**C**) and Trabecular number (**D**) Cortical wall thickness (**E-F**) were shown. All data are the mean ± SEM.*p < 0.05.

To investigate the regulation of CD44 on osteoclast function *in vivo*, we examined the effect of *CD44* deficiency on the decrease in bone formation in hindlimb-unloaded mice. The results of micro CT revealed that WT and *CD44* KO control mice showed a similar bone phenotype (Fig. 4A). In hindlimb-unloaded mice, trabecular bone volume (BV/TV) (Fig. 4B), trabecular thickness (Tb.Th) (Fig. 4C), and trabecular number (Tb.N^*^) (Fig. 4D) were decreased in WT and *CD44* KO mice. Cortical bone area and thickness were significantly decreased in WT mice after hindlimb unloading, however, there were no obvious changes in *CD44* KO mice (Fig. 4E,F). These results suggest that hindlimb unloading-induced changes in cortical bone were efficiently attenuated in *CD44* KO mice.

### CD44 inhibits osteoclast but not osteoblast function in hindlimb-unloading induced bone loss

**Figure 5:**
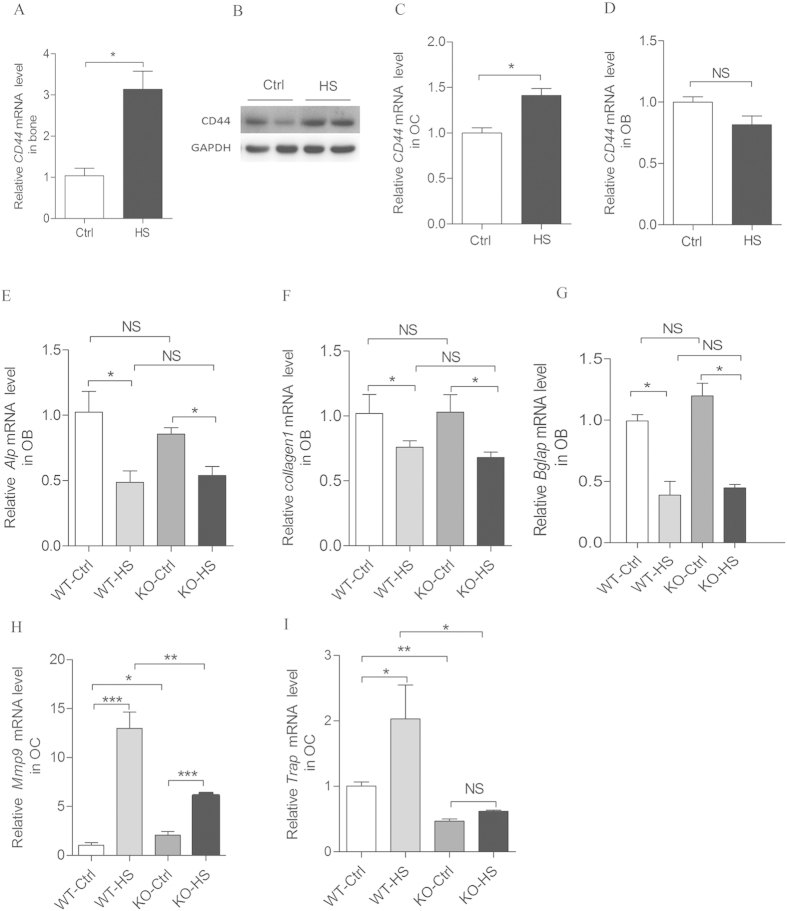
*CD44* deficiency reduces osteoclast funtion but not osteoblast in hindlimb-unloaded mice. The CD44 mRNA level (**A**) and protein level (**B**) in whole bone tissues collected from the hindlimb-unloaded and age-matched control mice were determined. qPCR analysis of CD44 mRNA levels in Alp^+^ (**C**) and Oscar^+^ (**D**) cells isolated by FACS from bone marrow stromal cells in bilateral tibias and femurs of hindlimb-unloaded and control mice. Real-time PCR analysis the expression of osteoblast marker genes, *Alp* (**E**), *Collagen I* (**F**) and *Bglap* (**G**) and osteoclast marker genes, *Mmp9* (**H**) and *Trap* (**I**)mRNA levels in tibias and femurs collected from WT-control (WT-Ctrl, n=6), WT-hindlimb-unloaded (WT-HS, n=6), *CD44* KO-control (KO-Ctrl, n=8) and KO-hindlimb-unloaded (KO-HS, n=8) mice. All data are the mean ± SEM. *p < 0.05, **p < 0.01, ***p < 0.001.

To assess the significance of CD44 in osteoclast or osteoblast function in the hindlimb-unloading mice model, we first analyzed changes in *CD44* mRNA expression levels in osteoblasts (Alp^+^) and osteoclast (Oscar^+^) cells from the hindlimb of tail-suspended and control mice. The results showed that *CD44* mRNA and protein levels increased in whole bone tissues and by 50% in osteoclasts after hindlimb-unloading, but there were no obvious changes in osteoblasts (Fig. 5A–D). The expression levels of osteoblast markers *Alp*, *Collagen-1*, and *Bglap* were all significantly decreased in bone tissues from WT and *CD44* KO mice 28 days after hindlimb unloading (Fig. 5E–G). However, the mRNA levels of osteoclast markers *Mmp9* and *Trap* had lower increase in the *CD44* KO group compared to WT mice (Fig. 5H, I). These results indicate that *CD44* deficiency inhibits cortical bone loss by inhibiting osteoclast function in the hindlimb unloading model.

## Discussion

**Figure 6:**
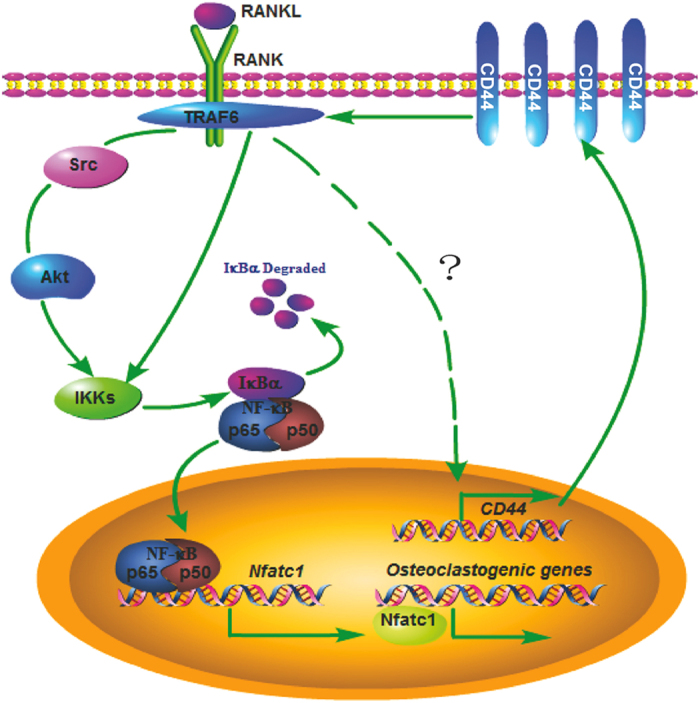
Model of CD44-mediated pathway in osteoclast differentiation and activity. After RANKL stimulation, the CD44 expression in osteoclast cells was upregulated. CD44 could increase the interaction between RANK and TRAF6, then it would activate its downstream signaling molecules, lead the phosphorylation of Src or Akt, which phosphorylates IκB-α and promotes the expression of NFATc1. NFATc1 induces the expression of genes related to the function and activity of osteoclast.

We demonstrated that the expression of *CD44* is upregulated during M-CSF- and RANKL-induced osteoclastogenesis. *CD44* KO resulted in a much lower expression of genes related to osteoclast differentiation and function *in vitro*. Osteoclasts induced from bone marrow-derived monocytes isolated from *CD44* KO mice exhibited reduced activity and function by TRAP staining and bone resorption measurement. We also found that CD44 was involved in the regulation of osteoclastogenesis through NF-κB-mediated signaling pathway. CD44 enhanced the interaction between RANK and TRAF6 and faciliates its down-stream signaling. In the hindlimb unloading model, the expression of CD44 was significantly upregulated in the hindlimb bone. *CD44* KO could protect from hindlimb unloading-induced cortical bone loss, whereby the downregulation of osteoclasts rather than osteoblasts contributed to this process. A model of the CD44-mediated pathway in osteoclast differentiation and activity is shown in Fig. 6.

CD44 is widely expressed in many tissues and cell types^35^. However, its expression and roles in skeletal tissues remain unclear. In this study, we found that the expression of CD44 was progressively up-regulated during M-CSF and RANKL-induced osteoclastogenesis. Osteoclasts originate from hematopoietic precursors of a monocyte/macrophage lineage and differentiate into multinucleated giant cells specialized to resorb bone by fusion of mononuclear progenitors^36^. RANKL interacts with the osteoclast cell surface receptor RANK, which in turn recruits TNF receptor associated factors (TRAFs) and plays a crucial role in osteoclast differentiation axis^37^. Our results demonstrated that CD44, as a membrane receptor, plays an important role in this process. Without CD44, the differentiation of BMMs into osteoclasts was greatly retarded and the function of osteoclasts was weakened.

Specific antibodies against CD44 inhibit osteoclast formation *in vitro*, thereby blocking the signal transduction of CD44 from extracellular matrix to intracellular regions. CD44 ligands such as HA and OPN inhibit BMM fusion *in vitro*^23^. The expression of NFATc1 is downregulated when HA binds to CD44, which leads to the downregulation of MMP-9, cathepsin K, and TRAP expression, as well as impairment of osteoclast migration and resorption activity^38^. In this study, we found that RANKL could induce the expression of CD44 during osteoclastogenesis. CD44 promotes the activation of RANKL-RANK-NF-κB-mediated signaling pathway by increasing the interaction between RANK and TRAF6.

The modulating role of CD44 in osteoclast formation depends on the microenvironment^39^. It has been reported that cancellous bone volume in the metaphysis of WT and *CD44* KO mice is normal. However, cortical thickness is increased and the medullary area is decreased in *CD44* KO mice^27^. In our experiments, we did not observe a difference in cortical thickness between WT and *CD44* KO mice. However, in the hindlimb unloading model, cortical bone loss was obviously alleviated in *CD44* KO mice. The expression of CD44 was significantly increased in the hindlimb bone of tail-suspended mice, which mainly resulted from the upregulation of CD44 in osteoclasts. After hindlimb unloading, the activity of osteoclast from *CD44* KO mice was much lower than that from WT mice. We also found that *CD44* expression is also up-regulated in femurs from ovariectomy (OVX) mice compared with control mice (see Supplementary Fig. S3A-C online). These results suggest that therapeutic inhibition of CD44 may protect from osteoporosis by inhibiting osteoclast activity.

It has been reported that OPN is regulated by mechanical stress *in vivo* and *in vitro*^40^,^41^. However, its mechanisms remain unclear. Our data demonstrated that CD44 is required for unloading-induced bone resorption *in vivo*, thereby suggesting that CD44 plays a key role in conveying the effect of mechanical stress to osteoclasts. Additional experiments are being performed to explore the OPN/CD44-mediated pathway in the regulation of osteoclast function under unloading-induced bone boss.

## Materials and Methods

### Animals

All WT and *CD44* knockout (KO) mice used in the experiments were bred and maintained at the SPF Animal Research Building of China Astronaut Research and Training Center (12-h light, 12-h dark cycles, temperature controlled for 23 °C and free access to food and water). Animals were fed with standard maintenance rodent diet (Beijing KEAO XIELI FEED Co. LTD, China). The mice used on this study were 4 month old males and in a C57BL/6J background. Mice were euthanized for dissecting bilateral femurs and tibias by injection with Avertin (2.5% 2,2,2-tribromoethanol; Sigma, USA). *CD44* KO mice were endowed by Dr. Li Tang from the Academy of Military Medical Sciences. The experimental procedures were approved by the Animal Care and Use Committee of China Astronaut Research and Training Center, and all animal studies were performed according to approved guidelines for the use and care of live animals.

### Micro-computed tomography (Micro-CT) analysis

High-resolution micro-CT analyses were performed on the distal femurs using a model of μ 40 scanco (Switzerland). In the femurs, the trabecular bone proximal to the distal growth plate was selected for analyses within a conforming volume of interest (cortical bone excluded) commencing at a distance of 840 μm from the growth plate and extending a further longitudinal distance of 1680 μm in the proximal direction. Cortical measurements were performed in the diaphyseal region of the femur starting at a distance of 3.57 mm from the growth plate and extending a further longitudinal distance of 210 μm in the proximal direction.

### Cell culture and osteoclast formation assay

Mouse bone marrow cells were isolated from the femur and tibia of 2-month-old mice. Briefly, bone marrow cells were flushed, collected and washed twice with α-MEM. Cells were then cultured with complete α-MEM medium in the presence of M-CSF (10 ng/ml, R&D, USA) for 1 day. Suspension cells were collected for osteoclast generation. Cells were cultured in complete medium with 30 ng/ml M-CSF and 50 ng/ml RANKL (R&D, USA) for 5 days. Tartaric acid phosphatase (TRAP) staining was according to the protocol of Acid Phosphatase kit (Sigma, USA). The TRAP positive multinuclear cells were recorded using inverted microscope (Nikon, Japan).

### Immunofluorescence

For immunostaining assay, mouse BMMs were cultured in complete medium with 30 ng/ml M-CSF and 50 ng/ml RANKL for 0, 3, 5 days. Then cells were washed three times with cold PBS and fixed in 4% Paraformaldehyde (Sigma, USA) for 30 min. After being washed three times with cold PBS, the cells were blocked at 37 °C for 1 h in 5% goat serum. Cells were incubated with anti-CD44 antibody (Abcam, USA) at room temperature for 2 h. After being washed three times with cold TBST (TBS, 0.1%Tween 20), the cells were incubated in goat anti-rabbit IgG/FITC at room temperature for 40 min. At last, the cells were incubated with DAPI (Roche, USA) for 15 min and then analyzed by confocal microscopy (Leica, Germany).

### RNA extraction and qPCR

Total RNA was extracted from cultured cells or bone tissue using RNAiso Plus reagent (Takara, China). The RNA was reverse transcribed into cDNA, and qPCR was performed using a SYBR Green PCR kit (Takara, China) in a Light Cycler (Eppendorf, Germany). The expression level of each gene was normalized to that of *Gapdh*, which served as an internal control. Primers (synthesized by Sunbiotech Co, China) for *CD44*, *Gapdh*, *CathepsinK*, *MMP9*, *Trap*, *CLC7*, *NFATc1*, *Alp*, *Bglap* and *Collagen1* were as follows:

### Pit formation assay

Mouse BMMs were obtained as described previously, for pit formation assay, BMMs (5 × 10^5^ cells/well) were seeded on bovine bone slices in 24-well plates in proliferation medium for 1 day and switched to differentiation medium for 3 days. Bovine bone slices were ultrasonicated in 1 mol/L NH_4_OH to remove adherent cells and stained with 0.1% toluidine blue solution^42^. Pit area was measured using Image Pro 405 Plus 6.2 software (Media Cybernetics Inc. USA).

### Western blot analysis

BMM cells were cultured with differentiation medium for 1, 3, 5 days. Cells were washed with cold PBS twice and then lysed in lysis buffer (50 mM Tris, pH7.5, 250 mM NaCl, 0.1% SDS, 2 mM dithiothreitol, 0.5% NP-40, 1 mM PMSF and protease inhibitor cocktail) on ice for 15 min. Cell extracts were collected by centrifugation at 15,00 0 g at 4 °C for 30 min, applied to 8–10% SDS-PAGE gels and transferred onto polyvinylidene difluoride (PVDF) membranes by electroblotting. The membranes were blocked for 1 hour in a blocking buffer containing 5% powdered milk in TBST. The membranes were incubated with primary antibody overnight at 4 °C followed by incubation with a secondary antibody conjugated to horseradish peroxidase (HRP), and visualized using an chemiluminescence kit (Thermo Pierce, No.32 109). Specific antibodies to p-Src (Cell Signaling Technology, #6943), Src (Cell Signaling Technology, #12945), p-Akt (Cell Signaling Technology, #9272), Akt (Cell Signaling Technology, #9271), p-IκBα (Cell Signaling Technology, #2859), IκBα (Cell Signaling Technology, #4814), NFATc1 (Cell Signaling Technology, #8032), NFκB (Santa Cruz Biotechnology, sc-7178), RANK (Santa Cruz Biotechnology, sc-9072), GAPDH (Santa Cruz Biotechnology, sc-25778) were used to detect protein levels.

### Immunoprecipitation

WT and CD44 knockout mouse BMMs were cultured in complete medium with 30 ng/ml M-CSF and 50 ng/ml RANKL for 5 days, and cells were harvested in in HEPES lysis buffer (20 mM HEPES pH 7.2, 50 mM NaCl, 0.5% Triton X-100, 1 mM NaF, 1 mM dithiothreitol) supplemented with protease inhibitor cocktail (Roche, Indianapolis, Indiana, USA) for immunoprecipitation. The cell lysates were transferred to a new fresh tube. Next 5 μg/ml rabbit polyclonal anti-TRAF6 (Abcam, USA) were added for 3 h and incubated the mixture with proteins A/G PLUS-agarose beads (Santa Cruz Biotechnology, USA) over night at 4 °C. Immune complexes were washed with cold lysis buffer for three times. After the final wash, we aspirated and discarded the supernatant and resuspended the pellet in 1X electrophoresis sample buffer and boiled it for 10 min.

### Cell sorting with flow cytometry

The bone marrow cells and bone marrow stromal cells were collected from the femur and tibia of 2-month-old WT and CD44 KO mice. Antibody to mouse Alp (R&D systems, Minneapolis, USA) and antibody to mouse Oscar (Santa Cruz Biotechnology, USA) antibodies were used for FACS according to the following protocol. After washed by PBS and 1% BSA, the cells were directly stained with antibody to Alp (1:50, R&D systems, Minneapolis, USA) and then stained with goat anti-mouse IgG-FITC (1:100, R&D systems, Minneapolis, USA) or were incubated with antibody to Oscar (1:40, Santa Cruz Biotechnology, sc-34237) and then stained with donkey anti-goat IgG-PE (1:100, R&D systems, Minneapolis, USA). After that, stained cell populations were used for FACS. The obtained selected Alp^+^ and Oscar^+^ cell populations were used for total RNA extraction and qPCR analysis.

### Hindlimb-unloading model

The hindlimb-unloading procedure was achieved by tail suspension, as described previously^43^. Briefly, the 4-month-old mice were individually caged and suspended by the tail using a strip of adhesive surgical tape attached to a chain hanging from a pulley. The mice were suspended at a 30° angle to the floor with only the forelimbs touching the floor, which allowed the mice to move and access food and water freely. The mice were subjected to hindlimb unloading through tail suspension for 28 d. After euthanasia, bilateral femurs and tibiae were dissected and processed for microCT examination and real-time PCR analysis.

### Statistical analysis

Data are presented as mean ± SEM per experimental condition. Considering the possibility of unequal variance for the data, we first test the equality of variances across groups. If it shows that the variances are unequal, we then use the Welch t test for 1-way analysis or mixed model with heterogeneous variances for 2-way analysis. Otherwise, we use the Student’s t test or the regular linear model. Bonferroni adjustment was used for multiple comparisons. p < 0.05 is considered statistically significant. p < 0.01 is considered very significant. All the statistical tests are analyzed by Prism software (Graphpad prism for windows, version 5.01) and SPSS (Version 14.0 for windows).

## Additional Information

**How to cite this article**: Li, Y. *et al.* *CD44* deficiency inhibits unloading-induced cortical bone loss through downregulation of osteoclast activity. *Sci. Rep.* **5**, 16124; doi: 10.1038/srep16124 (2015).

## Supplementary Material

Supplementary Information

## Figures and Tables

**Table 1:** 

*CD44*-F	5′-ACCATCGAGAAGAGCACC-3′
*CD44*-R	5′-TCATAGGACCAGAAGTTGTGG-3′
*Gapdh*-F	5′-TCACCACCATGGAGAAGGC-3′
*Gapdh*-R	5′-GCTAAGCAGTTGGTGGTGCA-3′
*Trap*-F	5′-GCGACCATTGTTAGCCACATACG-3′
*Trap*-R	5′-CGTTGATGTCGCACAGAGGGAT-3′
*Mmp9*-F	5′-GCTGACTACGATAAGGACGGCA-3′
*Mmp9*-R	5′-GCGGCCCTCAAAGATGAACGG-3′
*NFATc1*-F	5′-ACGCTACAGCTGTTCATTGG-3′
*NFATc1*-R	5′-CTTTGGTGTTGGACAGGATG-3′
*CathepsinK*- F	5′-GCGTTGTTCTTATTCCGAGC-3′
*CathepsinK*-R	5′-CAGCAGAGGTGTGTACTATG-3
*Clc7*-F	5′-GTCCTTCAGCCTCAGTCG-3′
*Clc7*-R	5′-ACACAGCGTCTAATCACAAC-3′
*Alp*-F	5′-ATCTTTGGTCTGGCTCCCATG-3′
*Alp*-R	5′- TTTCCCGTTCACCGTCCAC-3′
*Bglap*-F	5′- CCAAGCAGGAGGGCAATA-3′
*Bglap*-R	5′- TCGTCACAAGCAGGGTCA-3′
*Collagen1*-F	5′- GGGACCAGGAGGACCAGGAAGT-3′
*Collagen1*-R	5′- GGAGGGCGAGTGCTGTGCTTT-3′
