# Supplementary material for: CD44 deficiency inhibits unloading-induced cortical bone loss through downregulation of osteoclast activity
Source: Sci Rep. 2015 Nov 4;5:16124. doi: 10.1038/srep16124 (PMC4632082; doi:10.1038/srep16124)
Supplement: Supplementary Information [file srep16124-s1.pdf]

CD44 deficiency inhibits unloading-induced cortical bone loss through downregulation of osteoclast activity

Yuheng Li[1#]; Guohui Zhong[1#]; Weijia Sun[1]; Chengyang Zhao[1,2,‡]; Pengfei Zhang[2]; Jinping Song[1]; Dingsheng Zhao[1]; Xiaoyan Jin[1]; Qi Li[1] ; Shukuan Ling[1*] & Yingxian Li[1*]

[1]State Key Lab of Space Medicine Fundamentals and Application, China Astronaut Research and Training Center, Beijing, China.

[2]Key Laboratory of Molecular and Cellular Biology of Ministry of Education, College of Life Science, Hebei Normal University, Shijiazhuang, China.

[#] Yuheng Li and Guohui Zhong contributed equally to this work.

[‡]Current affiliation: College of Life Sciences, University of the Chinese Academy of Sciences, Beijing, China.

* Address for Correspondence:

Yingxian Li, Ph.D., State Key Lab of Space Medicine Fundamentals and Application, China Astronaut Research and Training Center, No. 26 Beiqing Road, Haidian District, Beijing, 100094, China. Tel: 8610-62895755, Fax: 8610-62895755, E-mail: yingxianli@aliyun.com or Shukuan Ling, Ph.D., State Key Lab of Space Medicine Fundamentals and Application, China Astronaut Research and Training Center, No. 26 Beiqing Road, Haidian District, Beijing, 100094, China. Tel: 8610-62895755, Fax: 8610-62895755, E-mail: sh2ling@126.com.

Materials and Methods

Stimulation with HA/OPN

In the presence of M-CSF(10 ng/ml), BMMs were cultured for one days, and then stimulated by HA (0.01%, $3.5x10^4$ Dalton, FREDA BIOPHARM, China) or OPN 5 ng/ml (R&D Systems, USA) for three days, and then cells were collected for western blot assay.

OVX-induced osteoporotic mouse

The procedure was described previously[1]. All the female C57BL/6J mice used were maintained under standard animal housing conditions (12-h light, 12-h dark cycles and free access to food and water). The mice were ovariectomized or sham-operated at 6 months of age. At 8 months after surgery (14 months of age), bilateral femurs from sham-operated and ovariectomized mice were collected for qPCR and western blot assay.

References:

1 **Wang, X. *et al.* miR-214 targets ATF4 to inhibit bone formation. *Nature medicine* 19, 93-100, doi:10.1038/nm.3026 (2013).**

Supplementary Figure1

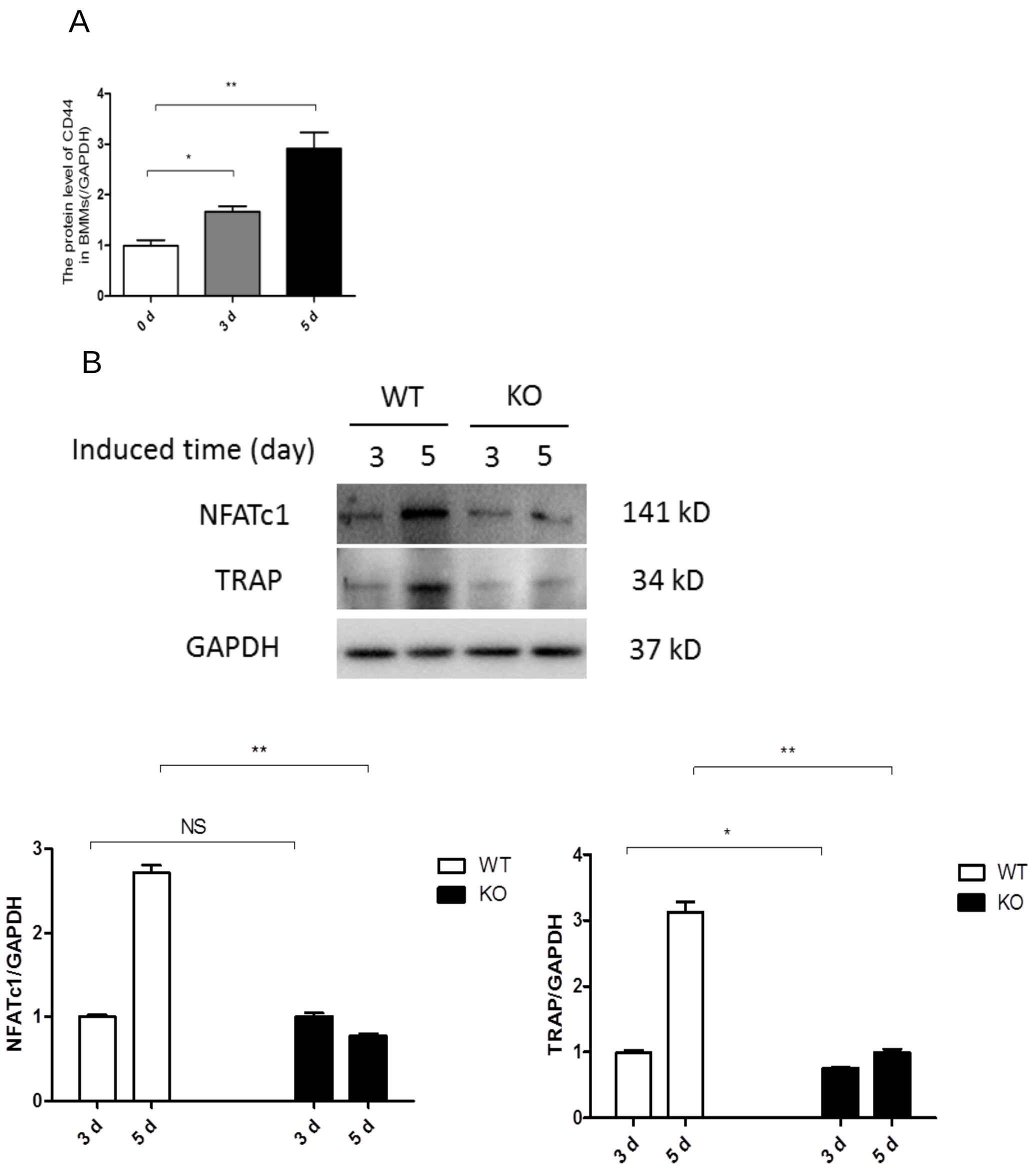

Supplementary Figure 1

(A) Densitometric analysis of CD44 protein levels (adjusted for GAPDH levels and expressed as the ratio to 0 day group) in all two groups using Image J software (version 1.45s for windows, NIH USA). Three independent experiments per group were performed, * $p < 0.05$, ** $p < 0.01$.

(B) The protein levels of TRAP and NFATc1 in WT and CD44 KO BMMs were analyzed by Western blotting.

Supplementary Figure 2

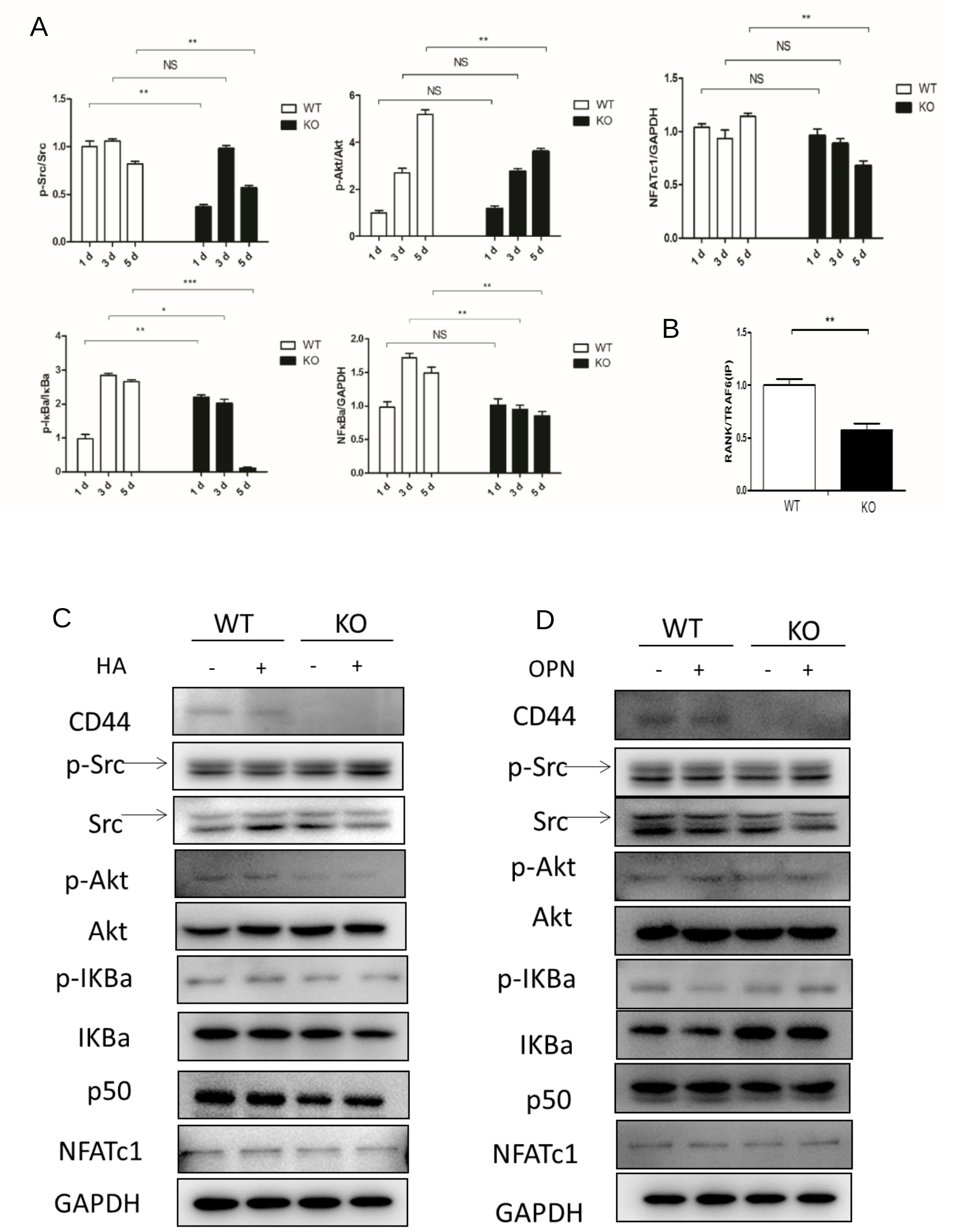

Supplementary Figure 2

(A) Densitometric analysis of p-Src (Tyr416) , p-Akt (S473), p-IκBα, NF-κB and NFATc1(adjusted for GAPDH levels and expressed as the ration to WT 1day group) in all groups using Image J software (version 1.45s for windows, NIH USA), Three independent experiments per group were performed, * $p < 0.05$, ** $p < 0.01$, *** $p<0.001$.

(B) Densitometric analysis of RANK immunoprecipitated by TRAF6 antibody (adjusted for TRAF6 levels in immunoprecipitated samples and expressed as the ration to WT group). Three independent experiments per group were performed, ** $p < 0.01$.

(C) And (D) BMMs from two-month-old WT and CD44 KO mice were cultured with HA (0.01%) and OPN (5ng/ml) for 3 days. Cell lysates were subjected to western blot analysis using specific antibodies. Representative western blot of CD44, p-Src (Tyr416) ,c-Src, p-Akt, Akt, p-IκBα, IκBα, NF-κB (p50) and NFATc1 were shown. n=3 per group.

Supplementary Figure 3

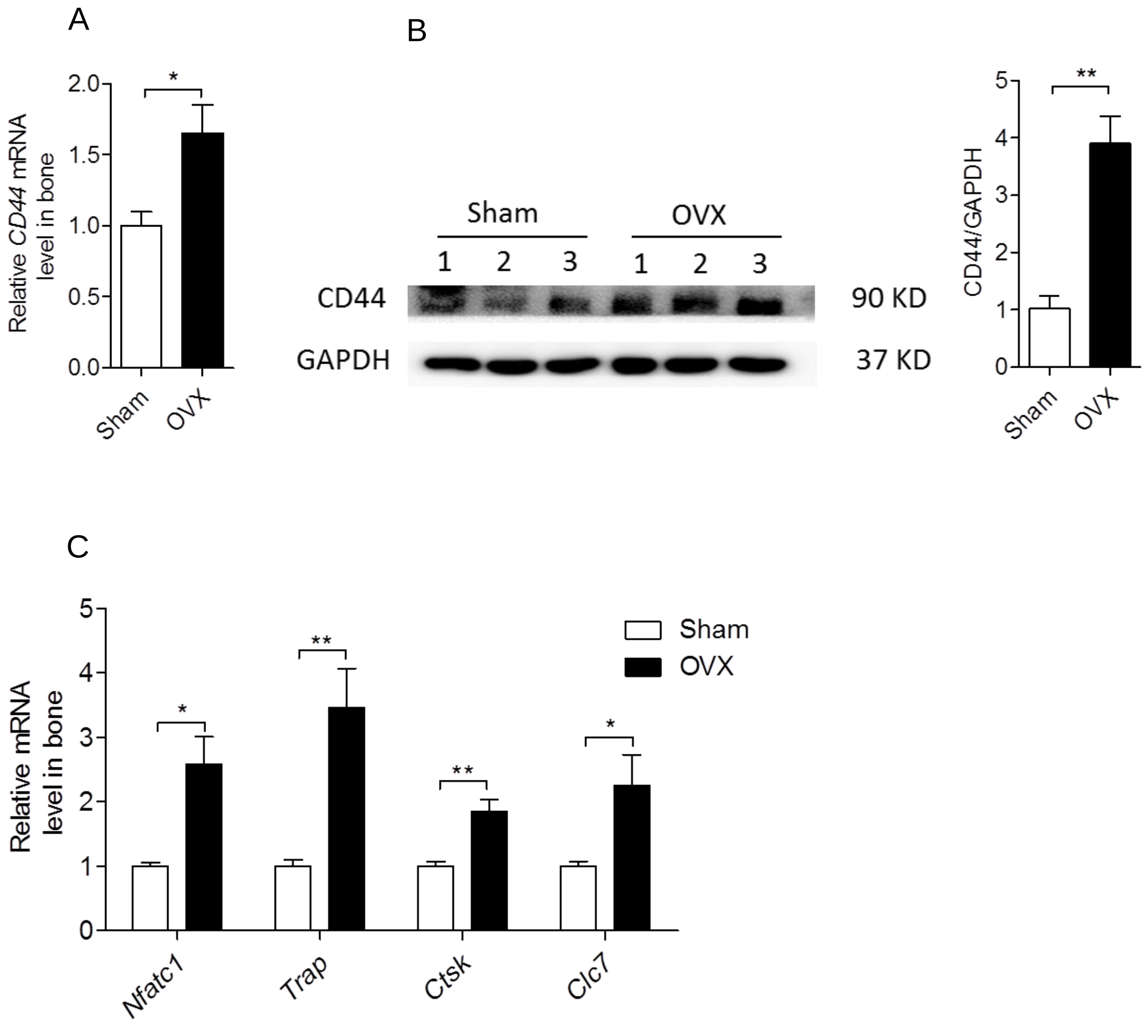

Supplementary Figure 3

CD44 mRNA (A) and protein (B) and osteoclast genes(*Nfatc1, Acp5, Ctsk* and *Clc7*) mRNA (C) levels in the femurs of mice receiving ovariectomy (OVX) or sham operation at 6 months of age collected at 8 months (14 months of age) after surgery.
